# The relationship between myasthenia gravis and COVID-19: a systematic review

**DOI:** 10.1186/s41983-022-00516-3

**Published:** 2022-07-07

**Authors:** Dodik Tugasworo, Aditya Kurnianto, Retnaningsih, Yovita Andhitara, Rahmi Ardhini, Jethro Budiman

**Affiliations:** Department of Neurology, Dr. Kariadi Hospital/Faculty of Medicine Diponegoro University, Dr. Sutomo 16, Semarang, 50244 Indonesia

**Keywords:** Autoimmune, COVID-19, Myasthenia gravis, Neurology

## Abstract

**Introduction:**

Viral infection such as coronavirus disease 2019 (COVID-19) can exacerbate and aggravate neurological disorders due to autoimmune etiology like myasthenia gravis (MG). Experimental therapies used in COVID-19 are also factors that can cause the worsening of MG symptoms. This review aimed to assess and conclude the research-based study systematically to analyze the relationship of MG and COVID-19.

**Method:**

This study was conducted in accordance to Cochrane handbook for systematic reviews and the guideline of preferred reporting items for systematic review and meta-analysis (PRISMA) and synthesis without meta-analysis (SWiM) in systematic reviews: reporting guideline. Inclusion criteria in this review were primary studies of every design, articles published in English around January 2000–October 2021, and the study used human as subject. A systematic literature finding was applied in 15 electronic scientific resources. The authors evaluated the study quality and risk of bias of each retrieved article.

**Results:**

The authors found the study through electronic scientific resources that met inclusion and exclusion criteria. The authors evaluated 362 articles identified in literature searching, 22 articles met the criteria for this review and then underwent the evaluation of study quality and risk of bias.

**Conclusion:**

COVID-19 infection can increase the risk of new-onset MG, myasthenic crisis, respiratory failure, and mortality rate due to cytokine storm in MG patients. The management of COVID-19 patients with MG is tailored to each person and based on national guidelines and local expert recommendations.

## Introduction

Coronavirus disease 2019 (COVID-19) is a novel infection due to severe acute respiratory syndrome coronavirus 2 (SARS-CoV-2) which can spread through droplet, aerosol, and contaminated objects [1–4]. The spreading of COVID-19 was increasingly widespread worldwide until the World Health Organization (WHO) on 9 March 2020 established COVID-19 as a global pandemic with a severity rate over 5% [5–8]. Until 2021 midyear, the prevalence of this disease was more than 170 million cases with a mortality rate about 2% [6]. These numbers will very likely increase for an unpredictable time with the progressing of the global pandemic dynamic.

Viral infections like COVID-19 can exacerbate and worsen neurological disorders caused by autoimmune etiology [9–16]. Myasthenia gravis (MG) is a neurological autoimmune disease due to autoantibody against the nicotinic acetylcholine receptor (nAChR). This blockade and downregulation of nAChR reduce nerve impulses that can generate muscle action potentials [17, 18]. In the COVID-19 pandemic, MG patients are at a greater risk of suffering COVID-19 and experiencing a poor outcome (when infected with COVID-19 compared to populations without this autoimmune condition). This occurs because of the immunocompromised status of MG patients due to immunosuppressant therapy, dysregulation of immune system, respiratory muscle weakness, and respiratory failure (because of pneumonia and pulmonary thromboembolism). On the other hand, COVID-19 infection has a great chance to trigger acute exacerbations in patients with MG because of the impairment of self-tolerance and activation of immune system, followed by increased T-cell signaling (and T-cell dysregulation induce autoantibody and autoimmunity) and the secretion of pro-inflammatory cytokines and molecules; inducing cytokine storm, acute respiratory distress syndrome (ARDS), and multi-organ failure; and also the theory about epitope spreading, bystander activation, immortalization of infected B cells, molecular mimicry (cross-reaction) [9–14, 16, 19]. Experimental therapies used in COVID-19 such as hydroxychloroquine and azithromycin are also factors that can cause worsening of MG symptoms due to the direct action on the neuromuscular junction [10, 12, 16, 20, 21]. ARDS due to COVID-19 in combination with respiratory muscle failure caused by MG may result in a dangerous condition; challenging for the clinician because of the increase of mortality rate in this combination. [11, 18]

Solé and colleagues (2021) reported that 0.96% of MG patients who registered in French database had COVID-19 infection [15]. Camelo-Filho and colleagues (2020) reported that 87% of patients with MG and hospitalized with COVID-19 were admitted to the intensive care unit, 73% used mechanical ventilation, and 30% (from all MG patients with COVID-19 in Camelo-Filho’s study) were deceased [10]. Patients with MG and COVID-19 have been presented in different studies, but a systematic review discussing this topic (with the publication type including observational studies) was not available. The guidelines for the management of MG patients in COVID-19 pandemic have been published but the recommendations are based on theory, not clinical data [22, 23]. The current systematic review aimed to assess and conclude the research-based study systematically to analyze the relationship of MG and COVID-19.

## Methods

This systematic review’s protocol was recorded on International prospective register of systematic reviews (PROSPERO) (CRD42021256169). This study was conducted in accordance to Cochrane handbook for systematic reviews and the guideline of preferred reporting items for systematic review and meta-analysis (PRISMA) [24, 25]. The data collection and analysis (synthesis) was also conducted based on synthesis without meta-analysis (SWiM) in systematic reviews: reporting guideline [26].

### Inclusion and exclusion criteria

Inclusion criteria in this review were full-text manuscripts reported the relationship of MG and COVID-19 and primary studies of every design (experimental study: clinical trial, observational study [descriptive study: case report and case series, and analytical studies: cross-sectional, case–control, and cohort]); articles published in English, articles published in January 2000—October 2021; the study used human as subject; and objective, methodology, and outcome of study must discuss the relationship of MG and COVID-19. Exclusion criteria were publication type was review and the study with variables that were associated in the relationship of MG and COVID-19.

### Literature search

A systematic literature finding was applied in these electronic scientific resources: Cambridge Core, Clinical Key, Cochrane, Ebsco, Embase, Emerald Insight, Google Scholar, JSTOR, Medline, Nature, Proquest, Pubmed, Science Direct, Scopus, and Springer Link. The search was performed using the following keywords for the title and abstract: (myasthenic OR myasthenia OR myasthenia gravis) AND (COVID-19 OR coronavirus OR SARS-CoV-2). The references from included studies were assessed as literature finding strategy.

### Data collection and analysis

Articles were chosen for assessment after two authors (DT and AK) had checked titles and abstracts from the electronic scientific resources. The results of the two authors were compared by a third author (R), and any differences of results were discussed. Selected full-paper were independently evaluated by the other authors (YA and RA). Selected articles for this systematic review were checked by two authors independently to confirm the results (AK and JB). The data from included articles were provided in a summary table featuring key points of each study. The key points of each study were: first author and country; study design; sample characteristic; management/outcome measure; and outcome/result.

### Quality assessment

The first author evaluated the study quality and risk of bias of each retrieved article and discussed them with other authors. Newcastle–Ottawa scale for cohort study was applied to evaluate the quality and risk of bias of cohort study; interpretation of total score was: ≥ 7 points were considered in good study, 5–6 points were considered in fair study, < 5 points were considered in poor study. Newcastle–Ottawa scale adapted for cross-sectional study was applied to evaluate the quality and risk of bias of the cross-sectional study. Interpretation of total score was: 9–10 points were included in very good study, 7–8 points were included in good study, 5–6 points were included in satisfactory study, and 0–4 points were included in unsatisfactory study [27–31]. The Joanna Briggs Institute (JBI) critical appraisal checklist was applied to evaluate the quality and risk of bias of descriptive study [32–34].

## Results

### Selection of articles for review

**Fig. 1:**
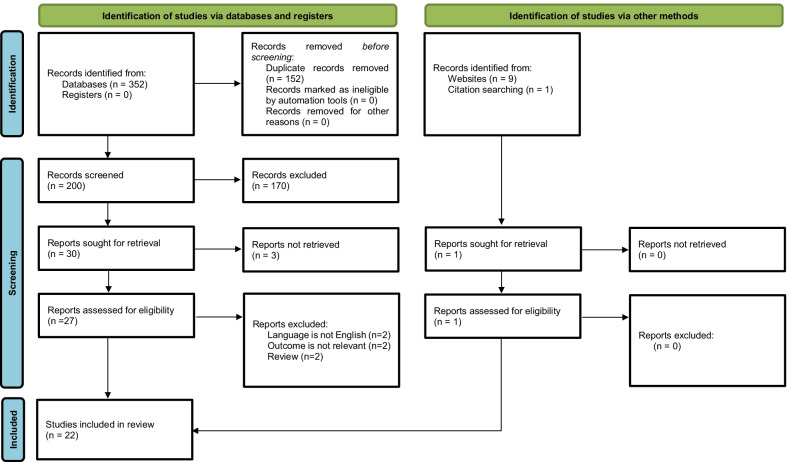
PRISMA flow diagram

Figure 1 provides PRISMA flow diagram. Initially, 352 peer-reviewed studies were found from electronic scientific resources and an additional 10 studies were identified through other sources (search engine). After duplicates were removed, 200 studies (titles and abstracts) were screened. Articles that did not meet the inclusion and exclusion criteria were not evaluated. Twenty-eight articles (27 articles from databases and registers, and 1 article from other methods) were screened for eligibility of which 22 articles were included in this review.

### Assessment of study validity (quality assessment and risk of bias)

All included studies were related to MG and COVID-19. Table 1 presents quality scores for cohort study and the studies had 6 points (fair studies). Table 2 presents quality scores for cross-sectional study and all of the studies included in very good, good and satisfactory studies. Tables 3 and 4 show JBI critical appraisal checklist for case report and case series, all of the studies got overall appraisal in “included studies” for systematic review.

**Table 1:** Newcastle–Ottawa scale (cohort study)

No.	First author, country	Selection				Comparability	Outcome			Total
		1	2	3	4		1	2	3	
1	Jakubíková M, Czech [35]	*		*	*		*	*	*	6
2	Kalita J, India	*		*	*		*	*	*	6
3	Sole G, French [15]	*		*	*		*	*	*	6

Maximum point for comparability was 2

Selection: (1) representativeness, (2) selection of non-exposed, (3) ascertainment of exposure, (4) demonstration that outcome was not present at the beginning

Outcome: (1) assessment of the outcome, (2) follow-up long enough, (3) adequacy of follow-up

**Table 2:** Newcastle–Ottawa scale adapted for cross-sectional study

No.	First author, country	Selection				Comparability	Outcome		Total
		1	2	3	4		1	2	
1	Businaro P, Italy [36]	*	*	*	**	**	*	*	9
2	Camelo-Filho AE, Brazil [10]	*	*	*	**		*		6
3	Stojanov A, Serbia [37]	*	*	*	**		*	*	7

Maximum points for selection number 4, comparability, and outcome number 1 were 2

Selection: (1) representativeness of the sample, (2) sample size, (3) non-respondents, (4) risk factor measurement tool

Outcome: (1) assessment of the outcome, (2) statistical test

**Table 3:** JBI critical appraisal checklist for case report

No.	Major components	1	2	3	4	5	6	7	8	9	10
1	Were patient’s demographic characteristics clearly described?	Y	Y	Y	Y	Y	Y	Y	Y	Y	Y
2	Was the patient’s history clearly described and presented as a timeline?	Y	Y	Y	Y	Y	Y	Y	Y	Y	Y
3	Was the current clinical condition of the patient on presentation clearly described?	Y	Y	Y	Y	Y	Y	Y	Y	Y	Y
4	Were diagnostic tests or assessment methods and the results clearly described?	Y	Y	Y	Y	Y	Y	Y	Y	Y	Y
5	Was the intervention(s) or treatment procedure(s) clearly described?	Y	Y	Y	Y	Y	Y	Y	Y	Y	Y
6	Was the post-intervention clinical condition clearly described?	Y	Y	Y	Y	Y	Y	Y	Y	Y	Y
7	Were adverse events (harms) or unanticipated events identified and described?	Y	Y	Y	Y	Y	Y	Y	Y	Y	Y
8	Does the case report provide takeaway lessons?	Y	Y	Y	Y	Y	Y	Y	Y	Y	Y
	Overall appraisal	I	I	I	I	I	I	I	I	I	I

1: Adhikari R, USA [38]; 2: Aksoy E, Turkey [39]; 3: Assini A, Italy [9]; 4: Essajee F, South Africa [40]; 5: Huber M, Germany [41]; 6: Moschella P, USA [42]; 7: Ramaswamy SB, USA [43]; 8: Reddy YM, India [19]; 9:Singh S, USA [44]; 10: Sriwastava S, USA [45]

*I* Include, *Y* Yes

**Table 4:** JBI critical appraisal checklist for case series

No.	Major components	1	2	3	4	5	6
1	Were there clear criteria for inclusion in the case series?	Y	Y	Y	Y	Y	Y
2	Was the condition measured in a standard, reliable way for all participants included in the case series?	Y	Y	Y	Y	Y	Y
3	Were valid methods used for identification of the condition for all participants included in the case series?	Y	Y	Y	Y	Y	Y
4	Did the case series have consecutive inclusion of participants?	Y	Y	Y	Y	Y	Y
5	Did the case series have complete inclusion of participants?	Y	Y	Y	Y	Y	Y
6	Was there clear reporting of the demographics of the participants in the study?	Y	Y	Y	Y	Y	Y
7	Was there clear reporting of clinical information of the participants?	Y	Y	Y	Y	Y	Y
8	Were the outcomes or follow-up results of cases clearly reported?	Y	Y	Y	Y	Y	Y
9	Was there clear reporting of the presenting site(s)/clinic(s) demographic information?	Y	Y	Y	Y	Y	Y
10	Was statistical analysis appropriate?	NA	NA	NA	NA	NA	NA
	Overall appraisal	I	I	I	I	I	I

1: Anand P, USA [46]; 2: Karimi N, Iran [47]; 3: Octaviana F, Indonesia [13]; 4: Peters BJ, USA [48]; 5: Saied Z, Tunisia [14]; 6: Zupanic S, Belgian [49]

*I* include, *NA* not applicable, *Y* yes

### Study characteristics

The study characteristics for the included studies could be seen in Tables 5 and 6. Sixteen studies were descriptive studies (10 case reports and 6 case series) and six studies were observational studies (three studies were cohort and three studies were cross-sectional). Most of the studies discussed about management and outcome of patient with COVID-19 and MG.

**Table 5:** Study characteristic of descriptive study

No.	First author, country	Study design	Subject characteristic	Management	Outcome
1	Adhikari R, USA [38]	Case report	33 y/o, M, AChR Ab, MG diagnosed before COVID-19	MV, steroid, symptomatic treatment	Deceased
2	Aksoy E, Turkey [39]	Case report	46 y/o, F, AChR Ab, MG diagnosed before COVID-19	Pyridostigmine (4 × 60 mg), favipiravir, meropenem, oseltamivir, HCQ (2 × 400 mg x 1d, 2 × 200 mg x 4d), MV, linezolid, MP iv (1 × 40 mg), and plasma therapy	Recovery
3	Anand P, USA [46]	Case series	MG diagnosed before COVID-19 1: 57 y/o, M, AChR Ab 2: 64 y/o, M, AChR Ab 3: 90 y/o, F, AChR Ab 4: 42 y/o, F, MuSK Ab 5: 64 y/o, F, AChR Ab	1: HCQ (2 × 400 mg x 1d, 1 × 200 mg × 2d), AZM (1 × 500 mg x 1d, 1 × 250 mg x 2d), TOZ (300 mg × 1 dose), AZA (1 × 50 mg), MV 2: HCQ (2 × 400 mg x 1d, 1 × 400 mg x 4d), AZM (1 × 500 mg x 1d, 1 × 250 mg x 4d), CTX (1 × 2 g x 2d, 1 × 1 g x 3d), prednisone (1 × 10 mg x 9d, 1 × 5 mg), MV 3: HCQ (2 × 400 mg x 1d, 1 × 400 mg x 4d), AZM (1 × 500 mg x 5d), CTX (1 × 1 g x 5 d), IVIG, prednisone (1 × 25 mg x 6d, 1 × 20 mg) 4: Prednisone (1 × 20 mg), IVIG (2 g/kg/d) 5: AZA, prednisone (1 × 60 mg)	1: Discharged home on day 9 2: Continued MV 3: Discharged to skilled nursing facility on day 19 4: Discharged home on day 5 5: Discharged home on day 9
4	Assini A, Italy [9]	Case report	77 y/o, M, MuSK Ab, newly diagnosed ocular MG triggered by COVID-19	Pyridostigmine (4 × 60 mg), AZA (1.5 mg/kg/d)	Recovery
5	Essajee F, South Africa [40]	Case report	7 y/o, F, AChR Ab, newly diagnosed ocular MG triggered by COVID-19	IV MP (30 mg/kg/d x 3d) → prednisone 2 mg/kg/d gradually weaned over 4 w, IVIG (2 g/kg/d x 2d), pyridostigmine, methotrexate	Discharge on day 30
6	Huber M, Germany [41]	Case report	21 y/o, F, AChR Ab, newly diagnosed ocular MG triggered by COVID-19	IVIG (0.4 g/kg/d x 5d), pyridostigmine (3 × 60 mg, increase to 3 × 120 mg next week)	Recovery
7	Karimi N, Iran [47]	Case series	Newly diagnosed ocular MG triggered by COVID-19 1: 61 y/o, F, AChR Ab 2: 57 y/o, M, AChR Ab 3: 38 y/o, F, AChR Ab	1: PE, pyridostigmine (4 × 60 mg), prednisone (1 mg/kg/d), thymoma surgery 2: pyridostigmine (3 × 60 mg), prednisolone (25 mg/d) 3: pyridostigmine (240 mg), prednisone (25 mg/d)	1: Recovery 2: Recovery 3: Recovery
8	Moschella P, USA [42]	Case report	70 y/o, M, AChR Ab, MG diagnosed before COVID-19	MV, hydrocortisone iv (100 mg), PE (5x), pyridostigmine (4 × 60 mg), methotrexate	Recovery
9	Octaviana F, Indonesia [13]	Case series	MG diagnosed before COVID-19 1: 25 y/o, F 2: 49 y/o, M 3: 42 y/o, F	1: Vit C (500 mg/d), NAC (600 mg/d), CTX (2 g/d), pyridostigmine (240 mg/d) → 6 d, AZM (500 mg/d x 1d) 2: AZM (500 mg/d), vit C (3000 mg/d), PCT (1500 mg/d), pyridostigmine (180 mg/d), AZA (100 mg/d)→ 5d 3: HCQ (200 mg/d), NAC (600 mg/d)→ 7 days, MP (16 mg/d), pyridostigmine (240 mg/d), mycophenolate (720 mg/d)	1: Discharge on day 14 2: Discharge on day 14 3: Discharge on day 14
10	Peters BJ, USA [48]	Case series	MG diagnosed before COVID-19 1: 71 y/o, M 2: 41 y/o, F 3: 59 y/o, M	1: Remdesivir (200 mg/d x 1d, 100 mg/d x 4d), dexamethasone iv (6 mg/d x 10d), lenzilumab (3 × 600 mg), mycophenolic acid 2: Remdesivir (200 mg/d x 1d, 100 mg/d x 4d), dexamethasone iv (6 mg/d x 10d), mycophenolate (1000 mg in morning, 1500 mg in evening), pyridostigmine (6 × 60 mg), prednisone after dexamethasone (1 × 5 mg) 3: Prone position, remdesivir (200 mg/d x 1d, 100 mg/d x 4d), dexamethasone iv (6 mg x 1d)→ prednisone 60 mg/d, AZA (100 mg in morning, 50 mg in evening), pyridostigmine (3 × 60 mg), MV	1: Deceased 2: Transferred out of the ICU 3: Discharged to home
11	Ramaswamy SB, USA [43]	Case report	42 y/o, F, AChR Ab, MG diagnosed before COVID-19	Prednisone (1 × 30 mg), mycophenolate (2 × 1000 mg)	Recovery
12	Reddy YM, India [19]	Case report	65 y/o, M, AChR Ab, newly diagnosed MG triggered by COVID-19	Remdesivir, IVIG (0,4 mg/kg/d x 5d), prednisolone (30–40 mg/d), AZT (2 × 50 mg), pyridostigmine (4 × 60 mg)	Discharge on day 23
13	Saied Z, Tunisia [14]	Case series	MG diagnosed before COVID-19 1: 40 y/o, F 2: 60 y/o, F 3: 37 y/o, F, AChR Ab 4: 57 y/o, M, AChR Ab 5: 54 y/o, F, AChR Ab	1: AZM (500 mg/d × 5 d), vit C (1000 mg/day x 10d), vit D (20,000 IU), LMWH x 10d 2: AZM (500 mg/d × 5 d), Vit C (1000 mg/day x 10d), vit D (20,000 IU x 10d), AZA (150 mg/d), pyridostigmine (8 × 60 mg) 3: AZM 500 mg/d × 5 d), vit C (1000 mg/day x 10d), vit D (20,000 IU/d) 4: MV, levofloxacin (500 mg/d), AZA (150 mg/d), pyridostigmine (8 × 60 mg), prednisone (40 mg/d) 5: AZM (500 mg/d × 5 d), Vit C (1000 mg/d × 10 d), Vit D (20,000 IU), LMWH × 10 d, AZA (150 mg), pyridostigmine (8 × 60 mg), IVIG (0.4 g/kg/d × 5 d)	1: Recovery 2: Recovery 3: Recovery 4: Deceased 5: Recovery
14	Singh S, USA [44]	Case report	36 y/o, F, negative AChR Ab and MuSK Ab, MG diagnosed before COVID-19	PE (5x), mycophenolate, MV, stress dose steroid iv	Discharged after 1 month, persistent anosmia
15	Sriwastava S, USA [45]	Case report	65 y/o, F, AChR Ab, newly diagnosed ocular MG triggered by COVID-19	Pyridostigmine (4 × 60 mg decrease to 3 × 60 mg when admitted to hospital again due to COVID-19 infection), dexamethasone iv (4 doses of 6 mg), azithromycin, 1 unit convalescent plasma	Discharged after 10 days with residual symptoms of COVID-10 and ocular MG
16	Zupanic S, Belgian [49]	Case series	MG diagnosed before COVID-19 1: 55 y/o, F, AChR Ab 2: 67 y/o, M, AChR Ab 3: 80 y/o, M 4: 63 y/o, M, AChR Ab 5: 59 y/o, F, negative Ab 6: 58 y/o, M, negative Ab 7: 51 y/o, M, AChR Ab 8: 66 y/o, F	1: IVIG (0,4 g/kg/d × 5 d), pyridostigmine (240 mg/d), AZA (100 mg), prednisolone (20 mg/d) 2: IVIG (0,4 g/kg/d x 5d), pyridostigmine (300 mg/d), prednisolone (60 mg/d) 3: Pyridostigmine (90 mg/d), remdesivir/5 d, dexamethasone (8 mg × 10 d) 4: IVIG (0,4 g/kg/d x 5d), pyridostigmine (360 mg/d), AZA (100 mg), prednisolone (60 mg/d), MV 5: Pyridostigmine (300 mg/d), dexamethasone (8 mg x 10d) 6: IVIG (0,4 g/kg/d x 5d), pyridostigmine (420 mg/d), prednisolone (30 mg/d), remdesivir/5d, MV 7: IVIG (0,4 g/kg/d x 1d), pyridostigmine (300 mg/d), remdesivir/5d 8: IVIG (0,4 g/kg/d x 5d), prednisolone (20 mg/d), remdesivir/5d, MV	1: Discharge on day 7 2: Discharge on day 12 3: Discharge on day 10 4: Discharge on day 16 5: Discharge on day 8 6: Discharge on day 15 7: Discharge on day 24 8: Deceased

**Table 6:** Study characteristic of observational study

No.	First author, country	Study design	Sample characteristic	Outcome measure	Result
1	Businaro P, Italy [36]	Cross-sectional	162 patients (11 patients had COVID-19 → 65 y/o, 54% M)	Outcome	3 patients needed MV and 2 patients died. 1 patient experienced worsening MG and improved after increasing steroid dose. COVID-19 patients significantly associated with MGFA ≥ III (p: 0,01)
2	Camelo-Filho AE, Brazil [10]	Cross-sectional	15 patients; 60% F (34.5 y/o), 40% M (61.3 y/o); 10 AChR Ab, 1 MuSK Ab	Outcome	87% admitted in the ICU, 73% needed MV, and 30% died
3	Jakubíková M, Czech [35]	Cohort	93 patients, 65.33 y/o, 51% M	Risk and protective factor	11% MG patients were dead due to COVID-19. Older age and long term use of steroid in MG patients were the risk factor of severe COVID-19 (p < 0.001, OR: 1.062, 95%CI: 1.037–1.088; p: 0.002, OR: 14.098, 95% CI: 1.784–111.43), while higher FVC before COVID-19 were protective factor of severe COVID-19 (p < 0.001, OR: 0.957, 95% CI: 0.934–0.98). Immunosuppressive drug (AZA, mycophenolate mofetil, and cyclosporine) were not associated in the worsening of COVID-19 (p: 0.8, OR: 1.147, 95% CI: 0.448–2.935; p: 0.1, OR: 3.375 95% CI: 0.91–12.515; p: 0.3, OR: 0.255, 95% CI: 0.029–2.212) and rituximab in MG patients increased the risk of death by COVID-19 (p: 0.004, OR: 35.143, 95% CI: 3.216–383.971). Remdesivir, favipiravir, and convalescent plasma were not associated with MG exacerbation (p: 0.4, OR: 1.709, 95% CI: 0.885–10.87)
4	Kalita J, India	Cohort	38 patients, 45 y/o, 42.1% F	QoL, daily living, anxiety and depression, and QoS of MG patients in COVID-19 pandemic	QoL, daily living, anxiety and depression, and QoS was impaired significantly in COVID-19 pandemic compared before pandemic (p < 0.05)
5	Sole G, French [15]	Cohort	3558 patients (0.96% had COVID-19 →55 ± 19.9 y/o, F: 55.9%)	Outcome	28 patients recovered from COVID-19, 1 remain affected, and 5 deceased. MGFA class ≥ IV was related with severe COVID-19 (p: 0.004)
6	Stojanov A, Serbia [37]	Cross-sectional	64 patients, 54.1 ± 16.4 y/o, 61.4% F	Psychological status, QoL, and QoS of MG patients in COVID-19 pandemic	Psychological status and QoL were impaired insignificantly, and QoS was reduced significantly in COVID-19 pandemic compared to 2017 (p < 0.01)

AChR Ab: acetylcholine receptor antibody

AZA: azathioprine

AZM: azithromycin

COVID-19: coronavirus disease 2019

CTX: ceftriaxone

d: day

F: female

HCQ: hydroxychloroquine

ICU: intensive care unit

iv: intravenous

IU: international unit

IVIG: intravenous immunoglobulin

LMWH: low molecular weight heparin

M: male

mg: milligram

MG: myasthenia gravis

MGFA: Myasthenia Gravis Foundation of America

MuSK Ab: muscle-specific tyrosine kinase antibody

MP: methylprednisolone

MV: mechanical ventilation

NAC; N-acetylcysteine

PE: plasma exchange

TOZ: tocilizumab

QoL: quality of life

QoS: quality of sleep

vit: vitamin

w: week

y/o: year old

## Discussion

### The relationship of MG and COVID-19

There were 10 descriptive studies focused in MG diagnosed before COVID-19 infection. The cohort study in French by Sole and colleagues (2021) showed that 0.96% of MG patients had COVID-19 with the mean age was 55 years, 55.9% was female, mean MG duration was 8.5 years, 26.5% had severe COVID-19, and mortality rate under 15%; while Businaro and colleagues (2021) study reported 11 COVID-19 patients from 163 MG patients in Italy [15, 36]. The risk factors of severe COVID-19 in study by Sole and colleagues (2021) based on univariate analysis were immunosuppressive drugs and MG severity (in multivariate analysis, only MG severity was related with poor outcome of COVID-19); in multivariate analysis the severity of MG patients/Myasthenia Gravis Foundation of America Classification (MGFA class ≥ IV) was related with severe COVID-19 (*p*: 0.004) [15]. The risk factor of severe COVID-19 in MG patients in Jakubíková M and colleagues (2021)’s study were older patients and long term use of steroid before COVID-19, and higher FVC was the protective factor. The interesting fact is that rituximab in MG patients increased the risk of COVID-19 death due to the failure of anti-SARS-CoV-2 antibody production (because rituximab acts as anti-CD20 monoclonal antibody, an important antibody to fight virus) [35]. The cross-sectional study by Camelo-Filho and colleagues (2020) reported that the COVID-19 patients with MG were 87% admitted in the ICU, 73% needed mechanical ventilation, and 30% died. This cross-sectional study also reported that risk factors for the mortality were male, geriatric, and had comorbidities [10]. COVID-19-associated risk and effects in MG (CARE-MG) reported that 40% of patients were in MG crisis and required emergency management [intravenous immunoglobulin (IVIG), plasma exchange (PLEX), or steroids], mortality rate was 24%, and 43% of patients discharged to home. [12]

COVID-19 in MG patients increased the risk of myasthenic crisis, respiratory failure, and permanent pulmonary damage. Molecular mimicry of SARS-CoV-2 and acetylcholine receptor and cytokine storm due to TNF-α, IFN-γ, IL-6, regulatory T cell (Th-17), and IL-17 is contributed to the ARDS in COVID-19 and myasthenic crisis and also associated with the severity, poor outcome, and the mortality [11, 14, 39]. There were 6 descriptive studies about new-onset of MG after COVID-19 infection. The similar structure from acetylcholine receptor and SARS-CoV-2 receptor, activation latent autoimmune disease, and hyperinflammation (such as multisystem inflammatory syndrome in children) may be the possible explanation of it [9, 19, 40, 41, 45, 47, 50]. This condition requires the use of mechanical ventilation, sedating, and paralytic drugs. The vital capacity under 20 mL/kg or negative inspiratory force under -20 cmH_2_O or forced vital capacity under 15 m/kg indicates respiratory failure and needs ventilator support [11, 51]. The use of mechanical ventilation must also consider the safety of the medical worker because of the risk of aerosolization transmission of the virus [46, 51]. The consideration use of non-invasive ventilation and biphasic positive airway pressure ventilation (BiPAP) also can be considered before intubation [11, 51]. The use of drugs like azithromycin and hydroxychloroquine may increase the risk of myasthenic disease or even myasthenic crisis, so must consider the benefit–risk ratio before using that drugs [46]. Octaviana and colleagues (2021) reported that the use of azithromycin and hydroxychloroquine were not increased the risk of deterioration in mild myasthenic patients with COVID-19, but the use of these drugs still requires local expert consideration due to the possible myasthenic flare in first 1 month after the first treatment [13, 23]. Peters and colleagues (2021) reported the beneficial use of remdesivir in MG and COVID-19. The pharmacodynamics of remdesivir is known to be not related to the acetylcholine receptor. [48]

The management of MG which involves immunosuppressive drugs like corticosteroid should be continued (with the consideration of local expert opinion and national guideline) [10, 52, 53]. A meta-analysis by van Paassen and colleagues (2021) reported the beneficial effect of corticosteroid on short-term mortality and the need for mechanical ventilation in COVID-19 patients due to the protective role (suppress the immune response of inflammatory cytokine) [52]. Sole and colleagues (2021) also reported that immunosuppressive drugs (like steroids) used for MG treatment were not related with poor outcome in COVID-19 patients nor protective effect [15]. Camelo-Filho and colleagues (2020) reported the beneficial effect of corticosteroids and immunosuppressive drugs which reduce the use of mechanical ventilation [10]. Saied Z and colleagues (2021) also described the good outcome of MG patients with COVID-19 who got corticosteroid [14]. The use of another immunosuppressive (mycophenolate mofetil) or immunomodulatory drug (IVIG or PLEX) in this condition must be considered case-by-case based on the benefit–risk ratio and the consideration of local experts [10, 22, 49, 54]. Jakubíková and colleagues (2021) reported that immunosuppressant drugs did not affect the worsening of COVID-19 due to the suppression of cytokine storm [35]. The use of immunosuppressive drug, targeted C5-complement inhibition (eculizumab) is also proved to be effective drug for MG and COVID-19 infection [55, 56]. Camelo-Filho and colleagues (2020) reported the good outcome with PLEX therapy and IVIG, and Zupanic and colleagues (2021) reported the beneficial use of IVIG in this case [10, 49]. The choice of PLEX or IVIG in the patient with COVID-19 and MG need special consideration from local expert, because PLEX has protective antibody and the mechanism to dispose of inflammatory cytokine, but PLEX removes both protective and harmful antibodies [42, 54]. Sriwastava and colleagues (2020) reported the continued use of pyridostigmine [45]. The results further confirm that the recommendation from the Guidance for the management of MG and LEMS during the COVID-19 pandemic by International MG/COVID-19 Working Group about the use of immunosuppressive drugs [22]. International MG/COVID-19 Working Group also recommends the adjustment of management according to each patient with underlying comorbidities by recommendation of expert, and continue standard MG management including pyridostigmine and eculizumab [22]. The use of pyridostigmine especially after intubation must need special attention because the effect of excessive airway secretion, and can be temporarily stopped if needed. [51]

Kalita and colleagues (2021) and Stojanov and colleagues (2020) reported the impact of COVID-19 pandemic on the quality of life and mental status of MG patients [37, 57]. The rapid transmission and the mortality rate of COVID-19 infection caused anxiety and depression in vulnerable people, including MG patients. This finding proved that professional therapeutic advice (from physician, psychologist, other medical workers, and the community) and psychosocial support are needed to reduce the stress especially in autoimmune diseases that need immunosuppressive therapy. The isolation precaution practice (standard and transmission practice: including hand hygiene, the use of personal protective equipment, and physical distancing) is also an important factor to reduce COVID-19 transmission. [22, 37, 57]

Advisory Committee on Immunization Practices (ACIP), American Association of Neuromuscular and Electrodiagnostic Medicine (AANEM), US Centers for Disease Control and Prevention (CDC), and International MG/COVID-19 Working Group recommend that MG patients can receive COVID-19 vaccine with the local physician recommendation (consider benefit–risk ratio and the attention of vaccination schedule based on patient’s condition and treatment due to the possibility of vaccine influencing immune response) and the consideration of best practice standard because the safety data of it are still in clinical trial [22, 58–62]. There were three case reports that discussed about the possibility exacerbation of moderate symptoms of MG because of COVID-19 mRNA vaccine in geriatric patients, but these reports need further follow-up and research related that incidences were causal or coincidental [63–65]. While Plymate and colleagues (2021) reported the safety of mRNA COVID-19 vaccine in MG patients and the benefit of additional doses of vaccine [66]. The Guidance for the management of MG and LEMS during the COVID-19 pandemic by International MG/COVID-19 Working Group suggests the use of dead vaccine in this group [22]. Ruan and colleagues (2021) reported the safety of inactivated COVID-19 vaccine (90.9% did not show MG symptoms in 1 month after vaccination and 9.1% had minor symptom but resolved quickly) [67]. The consideration of COVID-19 vaccination in MG patients is also based on the research that influenza vaccine is safe in MG patients. [59, 68, 69]

### Strength and limitation of the study

This systematic review consisted of 22 studies that explained the relationship of MG and COVID-19. The majority of the studies discussed the management and clinical outcome of patient with MG and COVID-19.

The limitation of the study was the most of study was descriptive study, the baseline characteristics were various, the variance of the demography in the human study, confounding variables in each study (human study), the lack of data of patients in outpatient settings, and limited follow-up time.

### Future implication

The current systematic review can be a scientific reading and material to physician, researcher, and all of the readers associated with the relationship of MG and COVID-19. Further research is needed with the larger sample size with diverse demographic variances and longer follow-up time; and also the marker of early detection of deterioration in MG and COVID-19.

## Conclusion

COVID-19 infection can increase the risk of new-onset myasthenia gravis, myasthenic crisis, respiratory failure, and mortality rate due to cytokine storm in myasthenia gravis patients. The management of COVID-19 patients with myasthenia gravis is tailored to each person and based on national guidelines and local expert recommendations.

## Data Availability

All data generated or analyzed during this study are included in this published article.

## Abbreviations

95% CI: 95% Confidence interval
AChR Ab: Acetylcoline receptor antibody
AZA: Azathioprine
AZM: Azithromycin
BiPAP: Biphasic positive airway pressure ventilation
CDC: US Centers for Disease Control and Prevention
COVID-19: Coronavirus disease 2019
CTX: Ceftriaxone
d: Day
F: Female
HCQ: Hydroxychloroquine
ICU: Intensive care unit
iv: Intravenous
IU: International unit
IVIG: Intravenous immunoglobulin
LMWH: Low molecular weight heparin
M: Male
mg: Milligram
MG: Myasthenia gravis
MGFA: Myasthenia Gravis Foundation of America
MuSK Ab: Muscle-specific tyrosine kinase antibody
MP: Methylprednisolone
MV: Mechanical ventilation
NAC: N-Acetylcysteine
nAChR: Nicotinic acetylcholine receptor
OR: Odds ratio
p: Probability
PE: Plasma exchange
TOZ: Tocilizumab
QoL: Quality of life
QoS: Quality of sleep
vit: Vitamin
w: Week
WHO: World Health Organization
y/o: Year old
